# Validation of the Korean Pediatric Emergency Tape with Two National Anthropometric Surveys in Korean Children

**DOI:** 10.3390/children12070913

**Published:** 2025-07-10

**Authors:** Dongbum Suh, Jin Hee Lee, Hyuksool Kwon

**Affiliations:** 1 Department of Emergency Medicine, Seoul National University Bundang Hospital, Seongnam 13620, Republic of Korea; dongbum@snubh.org (D.S.); jinuking3g@snubh.org (H.K.); 2 Department of Emergency Medicine, Seoul National University College of Medicine, Seoul 03080, Republic of Korea; 3 Research Center for Disaster Medicine, Seoul National University Medical Research Center, Seoul 03080, Republic of Korea

**Keywords:** child, heart arrest, overweight, resuscitation, weights and measures, emergencies, critical care

## Abstract

**Background**: The Korean Pediatric Emergency Tape (KPET), developed using 2005 anthropometric data, aims to improve weight estimation in Korean children. However, its validity has not been evaluated using recent large-scale data. This study evaluates the accuracy of the KPET compared with the latest version of the Broselow Tape (BT) using contemporary national anthropometric datasets. **Methods**: A cross-sectional analysis was conducted using pooled data from the 2019 National Health Screening Program for Infants and Children (NHSPIC, age 0–5) and the 2018–2019 Student Health Examination Sample Survey in Korea (SHESS, age 6–12). Accuracy was assessed by the proportion of estimates within 10% (PW10) and 20% (PW20) of measured weight, and by concordance between estimated and measured weight color zones. **Results**: Data from 1,992,646 (KPET) and 1,987,504 (BT) children were analyzed. In NHSPIC, the KPET showed slightly lower overall accuracy than the BT (PW10: 72.7% vs. 74.0%) but outperformed the BT in infants (PW10: 72.1% vs. 67.4%). In SHESS, the KPET consistently underperformed compared with the BT (PW10: 49.5% vs. 52.9%). The KPET showed higher concordance only in infants. Both tapes showed a trend of underestimation with increasing age, more pronounced in the KPET. **Conclusion**: The KPET showed lower overall performance than the BT but outperformed the BT in infants. Its accuracy declines in older children and tends to underestimate weight. Regular updates using recent anthropometric data are necessary to ensure accurate weight estimation and reflect current growth trends in Korean children.

## 1. Introduction

Rapid and accurate weight estimation is critical in pediatric emergency care, as it determines appropriate medication dosages, defibrillation energy levels, and the selection of correctly sized emergency equipment which are weight-dependent [1]. Length-based methods, adjusted for body habitus, provide the most accurate weight estimation of a child’s total body weight when actual weight is unavailable [2]. Consequently, both Advanced Trauma Life Support (ATLS) and Pediatric Advanced Life Support (PALS) guidelines recommend the use of body length tapes when a pediatric patient’s weight is unknown [1,3]. The Broselow Tape (BT), a measuring tape that estimates a child’s average weight based on height, is widely used for this purpose and has been globally validated for its effectiveness [4,5,6].

However, previous studies have shown that the BT accurately predicts the body weight of only about two-thirds of Korean children [7]. This discrepancy likely arises because the BT was developed using data from children in the United States. Recognizing this limitation, several countries have developed alternative weight estimation methods tailored to their pediatric populations’ unique anthropometric characteristics [8]. Although the BT has widespread international use, it has had limited availability in Korea, as there is no official distributor in the Korean market. This limited accessibility, combined with concerns about its accuracy for Korean children, prompted the Korean Association of Cardiopulmonary Resuscitation to develop and begin distributing the Korean Pediatric Emergency Tape (KPET) in 2018 using data from a 2005 nationwide anthropometric survey [9,10]. Currently, the KPET is actively promoted and taught in the Korean Pediatric Advanced Life Support courses and is widely integrated into medical education programs.

Despite its relatively recent introduction, the effectiveness of the KPET has not yet been fully validated for the current Korean pediatric population, with the only available study assessing its validity by comparing the endotracheal tube (ETT) sizes estimated using the KPET with the actual ETT sizes used in 1237 children [10]. However, since ETT size is determined in relatively large increments and is therefore not highly sensitive to small changes in body weight—and given that medication dosages and defibrillation energy require finer adjustments even for slight weight variations—it is more critical to directly compare the estimated weight with the actual measured weight to more accurately assess the validity of the KPET.

Since 2005, no comprehensive nationwide program has measured the height and weight of all Korean children and adolescents. However, two major anthropometric surveys provide relevant data for specific age groups: the National Health Screening Program for Infants and Children (NHSPIC) and the Student Health Examination Sample Survey in Korea (SHESS).

The NHSPIC is a national program designed to monitor the growth and development of infants and children aged 4 to 71 months in Korea [11]. It includes questionnaires, physical examinations, anthropometric measurements, health education, developmental assessments, and consultations. The NHSPIC is conducted in seven rounds: at 4–6 months, 9–12 months, 18–24 months, 30–36 months, 42–48 months, 54–60 months, and 66–71 months of age. In contrast, the SHESS is an annual sample survey conducted by the Korean Ministry of Education that assesses the health status of elementary, middle, and high school students (6 to 18 years of age) across approximately 1000 schools nationwide [12]. This survey collects data on physical development (including height and weight), health questionnaires, medical examinations, and laboratory tests.

Given that the KPET was developed using data collected nearly two decades ago, during which time the height, weight, and obesity rates of Korean children have continued to evolve, there is a clear need to reevaluate its current validity. Accordingly, the purpose of this study is to assess the accuracy and effectiveness of the KPET, in comparison with the previously used BT, using recent national anthropometric data from Korean children.

## 2. Methods

### 2.1. Study Design, Setting, and Population

This was a cross-sectional study involving a secondary analysis of pooled data from two national surveys: the NHSPIC (2019) and the SHESS (2018 and 2019). Data from 2018–2019 were selected as they represented the most recent comprehensive national datasets available at the time of study initiation. These data also represent the last period of standardized data collection under normal healthcare delivery conditions before the COVID-19 pandemic, which potentially affected screening procedures and data quality from 2020 onward despite maintained participation rates. While more recent data (2023) have since been released, they were not available during the planning and conduct of this research.

NHSPIC data were obtained through the National Health Insurance Data Sharing Service of the National Health Insurance Service, while SHESS data were sourced from publicly available datasets provided by the Ministry of Education.

The study included children aged 0–12 years. Data were excluded if any key study variables were missing or if the child’s height was outside the applicable ranges for both the KPET and BT. The height range for the KPET was 46.8–143.4 cm, and for the BT, 48.0–146.0 cm.

We measured the lengths of the weight sections on the KPET (published in December 2018; PanMun Education, Seoul, Republic of Korea) and the most recent edition available at the time of manuscript preparation, the 2019 edition, of the BT (Armstrong Medical Industries, Inc., Lincolnshire, IL, USA), using a steel ruler (Komelon Corporation, Busan, Republic of Korea).

### 2.2. Methods and Measurement

(1) Data extraction and study procedure

Data for children aged 0 to 5 years were extracted from the NHSPIC, while data for children aged 6 to 12 years were obtained from the SHESS. From the NHSPIC dataset, we collected information on sex, the round number of screening programs, weight, and height. Since the dataset did not contain age or any date-related variables, the round number of the screening programs was used as a proxy for age. Thus, children in rounds 1 and 2 were categorized as 0 years of age, round 3 as 1 year, round 4 as 2 years, round 5 as 3 years, round 6 as 4 years, and round 7 as 5 years. If a participant appeared multiple times, only the first record was included. From the SHESS dataset, we extracted data on sex, date of birth, date of examination, weight, and height. Age was calculated based on the time elapsed from the date of birth to the date of examination.

In both datasets, the estimated weights and color-coded zones were determined by applying each child’s measured heights to the corresponding intervals on both the KPET and the BT.

(2) Outcomes

The primary outcomes were (1) the accuracy of the KPET and BT weight estimates, defined as estimates within 10% and 20% of the measured weight (PW10 and PW20, respectively), and (2) the concordance between color-coded zones derived from the tape’s estimated weights and the zones based on measured weights.

(3) Statistical analysis

Continuous variables are presented as means and standard deviations, while categorical variables are expressed as percentages. The accuracy of the tapes was determined by calculating PW10 and PW20. Bias and precision were further assessed using the mean percentage error (MPE), mean absolute percentage error (MAPE), and root mean square error (RMSE). Agreement between estimated and measured weights was examined using Bland–Altman analysis, with differences expressed as percentages to determine the limits of agreement (LOAs). The 95% LOAs of the mean percentage error (PELOA) were calculated for each age group to quantify precision. Percentage differences were reported instead of absolute differences to minimize bias, as absolute errors tend to increase as body weight increases.

Additionally, we compared the color-coded zones determined from estimated weights with those based on measured weights. Each color-coded zone was assigned a numerical value, allowing overall concordance to be calculated.

*p*-values < 0.05 were considered statistically significant. All statistical analyses were performed using Stata/SE version 18 (StataCorp LLC, College Station, TX, USA).

## 3. Results

### 3.1. Patient Characteristics

**Figure 1:**
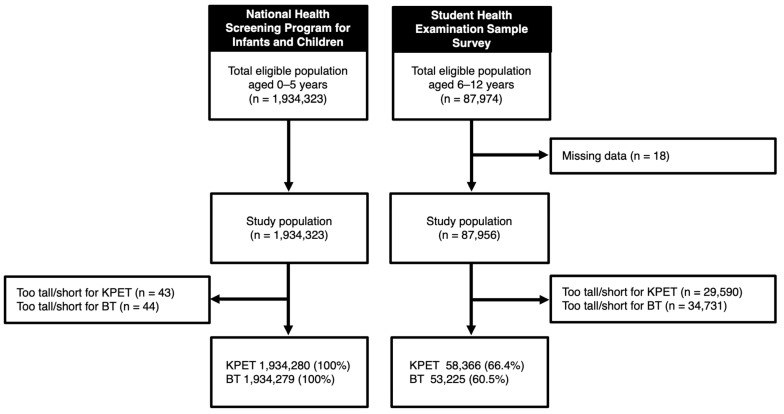
Study population. *KPET*, Korean Pediatric Emergency Tape; *BT*, Broselow Tape.

A total of 2,022,279 children were included in the analysis, of whom 1,992,646 (98.5%) were evaluated for the KPET, and 1,987,504 (98.3%) for the BT (Figure 1). The mean age in the NHSPIC population was 2.8 years (standard deviation [SD] 1.7), and in the SHESS population, 9.8 years (SD 2.0). Boys accounted for 51.3% of the NHSPIC population and 52.2% of the SHESS population. Detailed characteristics of the study population are presented in Table 1.

### 3.2. Accuracy, Bias and Precision of the Weight-Estimating Tapes

The accuracy of weight estimation using both tapes, assessed by PW10 and PW20, is summarized in Table 2. Statistical comparisons showed significant differences between KPET and BT performance across all age groups (all *p* < 0.05). In the NHSPIC population (children aged 0–5 years), the KPET demonstrated lower accuracy overall, with a PW10 of 72.7%, compared to 74.0% for the BT (*p* < 0.05). For PW20, the KPET had 96.2%, while the BT had 96.6% (*p* < 0.05). However, among infants aged 0 years, the KPET achieved higher PW10 and PW20 than the BT (PW10: 72.1% vs. 67.4%, *p* < 0.05; PW20: 97.2% vs. 95.9%, *p* < 0.05). In the SHESS population (children aged 6–12 years), the KPET consistently showed lower PW10 and PW20 accuracy compared to the BT (PW10: 49.5% vs. 52.9%, *p* < 0.05; PW20: 80.7% vs. 84.1%, *p* < 0.05).

**Figure 2:**
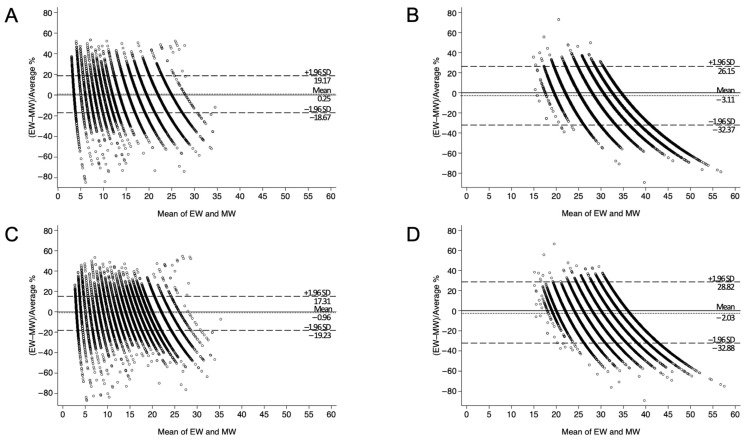
Bland–Altman plots comparing tape-estimated weight (EW) and measured weight (MW) using percentage error. (**A**) KPET-eligible children aged 0–5 years. (**B**) KPET-eligible children aged 6–12 years. (**C**) BT-eligible children aged 0–5 years. (**D**) BT-eligible children aged 6–12 years. *SD*, standard deviation; *KPET*, Korean Pediatric Emergency Tape; *BT*, Broselow Tape.

**Figure 3:**
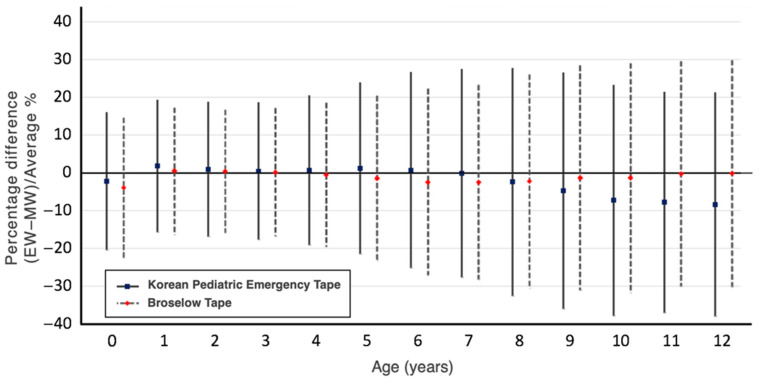
Bias and limits of agreement between tape-estimated weight (EW) and measured weight (MW) using percentage error by age.

Regarding bias and precision, the KPET showed lower bias and higher precision among infants aged 0 years, while the BT showed lower bias and higher precision in children aged 8 years and older (Table 2). Bland–Altman plots using percentage error revealed that, in the NHSPIC population, the KPET had a lower mean bias, while the BT showed narrower PELOA. In the SHESS population, the KPET showed a higher mean bias but a narrower PELOA (Figure 2). When analyzed by age, the KPET demonstrated both lower bias and a narrower PELOA in infants aged 0 years (KPET: bias −2.28, PELOA [−20.56, 16.00]; BT: bias −3.91, PELOA [−22.61, 14.80]) (Figure 3). However, as children got older, the KPET exhibited progressively greater bias, particularly in those aged 8 years and above.

### 3.3. Concordance of Color-Coded Zone

**Figure 4:**
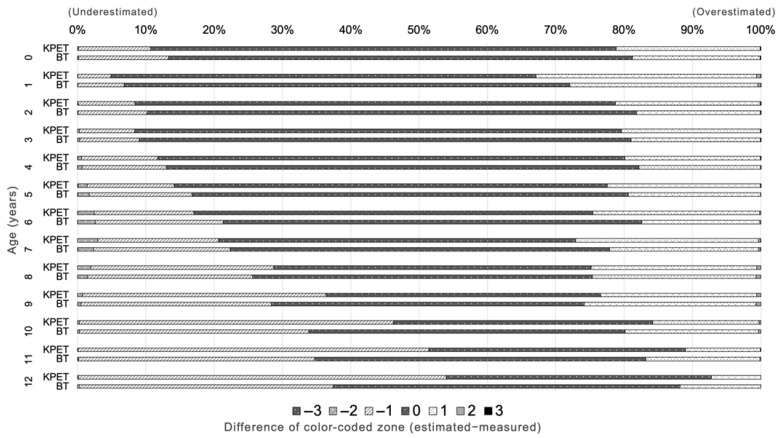
Concordance of color-coded zones between tape-estimated weight and measured weight. *KPET*, Korean Pediatric Emergency Tape; *BT*, Broselow Tape.

Figure 4 illustrates the concordance of color-coded zone by age. In the NHSPIC population, the concordance was 67.6% for the KPET and 68.5% for the BT (*p* < 0.05), while in the SHESS population, it was 46.1% for the KPET and 47.0% for the BT (*p* < 0.05). The KPET showed higher concordance only among infants aged 0 years, whereas the BT outperformed the KPET in all other age groups. Notably, the trend toward underestimation of weight became more pronounced with age in both tapes, but especially for the KPET.

## 4. Discussion

This study validates the KPET, a length-based weight estimation tool developed and utilized in Korea, by comparing it with the widely used BT using national-level height and weight data from South Korea. The results indicate that the KPET had an accuracy within 10% for approximately 73% of children under the age of 5 and about 50% of children aged 6 and older. Notably, the KPET demonstrated superior performance and accuracy compared to the BT in children aged 0 years. However, for other age groups, the performance and accuracy of the KPET were either comparable to or inferior to those of the BT. Notably, in children aged eight years and older, the accuracy of the KPET declines progressively with increasing age, suggesting its reduced reliability in older pediatric populations.

Korean infants (under 30 months) tend to have greater height and weight compared to their Western counterparts [13,14]. This difference in physical characteristics may explain why the KPET, which was developed based on data from Korean children and reflects the unique growth patterns and health metrics of the Korean population, demonstrated superior performance compared to the BT in the infant group. However, the KPET showed a tendency to underestimate actual weight in older pediatric age groups compared to the BT. This discrepancy may be attributed to the higher obesity rate among Korean children over the age of five, which has been increasing rapidly in recent years [15]. While the recently updated BT has addressed this issue by incorporating more recent data, the KPET, developed using data from over two decades ago, may not fully reflect these changes.

### 4.1. Weight Accuracy Versus Color Zone Concordance

The observed discrepancy between relatively high PW10/PW20 accuracy rates and lower color-coded zone concordance can be explained by the discrete nature of color-coded weight estimation systems. Color-coded systems like the KPET and the BT provide only a single representative weight value per color zone, creating discrete boundaries between adjacent zones. When a child’s actual weight falls near the boundary between adjacent color zones, even small estimation errors that remain within acceptable weight accuracy ranges (±10–20%) can result in classification into a different color zone.

This explains why continuous weight-based accuracy metrics (PW10/PW20) can appear favorable while discrete color zone concordance remains lower. Previous studies have documented similar findings, with research showing substantial discrepancies between weight estimation accuracy and color zone placement [16]. Other studies have reported that while weight estimation tapes were inaccurate in predicting the correct color zone in substantial proportions of children, the majority of discrepancies involved only one color zone. The findings of this study are consistent with those observations [17].

From a practical emergency care perspective, these findings suggest that for weight-dependent interventions such as medication dosing and defibrillation energy, clinicians may consider using the estimated weight value directly rather than relying solely on color zone classifications. For equipment selection, color zones can still serve as a helpful guide. However, when the estimated weight falls near a zone boundary, clinical judgment based on factors such as the child’s body habitus and overall appearance may help ensure the appropriate equipment choice. The future development of weight estimation tools may benefit from incorporating strategies that enhance both accuracy and clinical applicability.

### 4.2. Clinical Implications and Practical Considerations

A key strength of this study is the utilization of a large-scale dataset that represents the Korean pediatric population. Specifically, the NHSPIC dataset includes over 70% of all Korean children aged 0–5 years, enabling a more reliable assessment of the KPET’s performance in this age group. Furthermore, given the current lack of large-scale, standardized pediatric anthropometric surveys, this study highlights the potential use of annually published datasets such as the NHSPIC and the SHESS for updating the KPET. These datasets could serve as valuable resources for improving the accuracy of weight estimation and ensuring that the KPET reflects the latest growth trends in Korean children.

The development and implementation of the KPET addresses a critical gap in pediatric emergency care in Korea. While the BT showed superior overall performance in older children, its limited official availability in Korea has historically restricted widespread adoption. In contrast, the KPET is actively distributed and promoted through national medical education programs, including pediatric advanced life support training. Our findings suggest that the KPET’s superior performance in infants (age 0 years) provides meaningful clinical value, particularly given Korea’s unique infant anthropometric characteristics. The comparable performance in children aged 1–5 years, combined with the KPET’s greater accessibility and integration into Korean medical education, supports its continued use while highlighting the need for performance improvements in older children. Among children aged 6 years and older, subtracting approximately 1 kg from KPET-estimated weights led to a meaningful improvement in accuracy (see Supplementary Table S1). This simple correction may enhance the clinical applicability of the KPET in older pediatric populations and serve as a practical interim strategy until an updated version of the tape is developed.

Despite the advantages of the KPET in specific age groups, this study underscores the need for an updated version of the KPET that reflects the current growth trends of Korean children. Future research should focus on revising the weight estimation tape using recent anthropometric data and incorporating additional factors, such as body habitus, to enhance its accuracy.

### 4.3. Limitations

This study has several limitations. First, although the 2018–2019 data may not represent the most current timepoint, they provide a substantial 13–14-year advancement from the 2005 data originally used to develop the KPET and represent data collected under standardized and uninterrupted healthcare conditions before the COVID-19 pandemic. Although the pandemic did not significantly reduce screening participation, potential concerns about data consistency during this period include changes in healthcare delivery practices and measurement environments. Nevertheless, future validation studies should incorporate newer datasets, including post-pandemic data, to assess both contemporary anthropometric trends and any potential pandemic-related effects on pediatric growth patterns. Second, this study utilized two independent datasets, the NHSPIC and the SHESS, which differ in age coverage and data collection approach, potentially introducing structural bias. The NHSPIC dataset, based on national health screenings, may underrepresent medically vulnerable children who are unable to participate in routine examinations, while the SHESS dataset, being sample-based, may be subject to sampling variability. Although direct comparison between the datasets was not appropriate, separate analyses within each consistently demonstrated statistically significant differences in the performance of the KPET and the BT across all age groups, thereby reinforcing the validity of the findings. Third, both the KPET and the BT estimate weight based on height alone, which does not account for variations in body habitus. The discrepancy between weight accuracy and color zone concordance represents an inherent limitation of all color-coded weight estimation systems rather than a specific weakness of the tapes evaluated. Future research should consider incorporating body mass index or other body composition parameters to improve the predictive accuracy of weight estimation tools and might explore adaptive zone boundaries or probabilistic approaches to equipment selection that account for estimation uncertainty.

## 5. Conclusions

This study assessed the accuracy and effectiveness of the KPET in estimating the weight of Korean children using national-level height and weight data. The results indicate that the KPET outperforms the widely used BT in infants aged 0 years; however, its accuracy declines in older children, particularly those aged eight years and older, with a tendency to progressively underestimate weight. While the KPET remains a valuable tool for pediatric emergency care in Korea, its performance is suboptimal in older children. Therefore, it may be most appropriate for use in children aged 5 years and younger, while caution is advised in older age groups. In this study, a reduction of approximately 1 kg from KPET-estimated weights appeared to improve accuracy in children aged 6 years and above, which may serve as a useful reference for clinical judgment. Given the increasing prevalence of childhood obesity and the evolving growth patterns of Korean children, an updated version of the KPET is needed to enhance its clinical utility and reliability in emergency settings.

## Figures and Tables

**Table 1:** Characteristics of study population.

Dataset	Age	Total	Eligible for Korean Pediatric Emergency Tape							Eligible for Broselow Pediatric Emergency Tape						
		*n*	*n* (%)		Boy (%)	Weight, Mean (SD)		Height, Mean (SD)		*n* (%)		Boy (%)	Weight, Mean (SD)		Height, Mean (SD)	
National Health Screening Program for Infants and Children	0	432,853	432,810	(100)	51.5	8.8	(1.3)	70.3	(4.9)	432,809	(100)	51.5	8.8	(1.3)	70.3	(4.9)
	1	289,890	289,890	(100)	51.5	12.1	(1.4)	86.0	(3.4)	289,890	(100)	51.5	12.1	(1.4)	86.0	(3.4)
	2	327,736	327,736	(100)	51.2	14.3	(1.7)	94.2	(3.5)	327,736	(100)	51.2	14.3	(1.7)	94.2	(3.5)
	3	330,542	330,542	(100)	51.2	16.4	(2.1)	101.2	(3.9)	330,542	(100)	51.2	16.4	(2.1)	101.2	(3.9)
	4	297,351	297,351	(100)	51.2	18.7	(2.8)	108.0	(4.3)	297,351	(100)	51.2	18.7	(2.8)	108.0	(4.3)
	5	255,951	255,951	(100)	51.3	21.3	(3.6)	114.3	(4.6)	255,951	(100)	51.3	21.3	(3.6)	114.3	(4.6)
	0–5	1,934,323	1,934,280	(100)	51.3	14.7	(4.7)	93.6	(15.7)	1,934,279	(100)	51.3	14.7	(4.7)	93.6	(15.7)
Student Health Examination Sample Survey	6	7757	7756	(100)	51.6	24.1	(4.8)	120.3	(4.9)	7755	(100)	51.6	24.1	(4.8)	120.2	(4.9)
	7	13,032	13,024	(99.9)	51.2	26.9	(5.8)	125.3	(5.4)	13,013	(99.9)	51.2	26.9	(5.7)	125.2	(5.3)
	8	12,806	12,700	(99.2)	50.8	30.8	(6.8)	131.1	(5.6)	12,501	(97.6)	50.6	30.5	(6.5)	130.9	(5.3)
	9	12,199	11,218	(92.0)	51.6	34.0	(7.3)	136.1	(5.1)	10,279	(84.3)	51.4	33.2	(6.8)	135.3	(4.7)
	10	13,319	8990	(67.5)	53.9	36.4	(7.2)	139.6	(4.2)	6988	(52.5)	53.4	35.1	(6.6)	138.2	(3.7)
	11	12,734	3711	(29.1)	56.3	37.3	(6.8)	141.6	(3.6)	2232	(17.5)	57.2	35.5	(6.3)	139.6	(3.2)
	12	16,109	967	(6.0)	61.4	38.0	(7.1)	142.8	(2.8)	457	(2.8)	63.5	36.1	(6.4)	140.6	(2.5)
	6–12	87,956	58,366	(66.4)	52.2	31.1	(7.9)	131.5	(8.6)	53,225	(60.5)	51.8	30.1	(7.2)	130.2	(7.9)

SD, standard deviation.

**Table 2:** Performance of the Korean Pediatric Emergency Tape and the Broselow Tape.

Datasets	Age	Korean Pediatric Emergency Tape					Broselow Tape				
		PW10, % (95% CI)	PW20, % (95% CI)	MPE (SD)	MAPE (SD)	RMSE (SD)	PW10, % (95% CI)	PW20, % (95% CI)	MPE (SD)	MAPE (SD)	RMSE (SD)
National Health Screening Program for Infants and Children	0	72.1 (72.0 to 72.2)	97.2 (97.2 to 97.3)	−2.28 (9.14)	7.40 (5.83)	0.66 (0.54)	67.4 (67.2 to 67.5)	95.9 (95.9 to 96.0)	−3.91 (9.35)	8.03 (6.18)	0.71 (0.58)
	1	76.3 (76.1 to 76.4)	97.5 (97.5 to 97.6)	1.79 (8.76)	6.99 (5.57)	0.84 (0.69)	78.4 (78.3 to 78.6)	98.3 (98.2 to 98.3)	0.50 (8.43)	6.60 (5.28)	0.80 (0.67)
	2	75.7 (75.6 to 75.9)	97.2 (97.2 to 97.3)	0.94 (8.92)	7.06 (5.53)	1.01 (0.84)	78.5 (78.4 to 78.7)	98.3 (98.2 to 98.3)	0.44 (8.21)	6.42 (5.14)	0.92 (0.80)
	3	75.3 (75.1 to 75.4)	97.1 (97.0 to 97.2)	0.43 (9.11)	7.05 (5.79)	1.17 (1.05)	78.3 (78.2 to 78.4)	97.8 (97.8 to 97.9)	0.16 (8.56)	6.52 (5.54)	1.09 (1.03)
	4	71.5 (71.3 to 71.6)	95.4 (95.3 to 95.4)	0.65 (9.93)	7.66 (6.35)	1.48 (1.43)	74.5 (74.3 to 74.7)	96.0 (95.9 to 96.0)	−0.49 (9.60)	7.29 (6.26)	1.43 (1.49)
	5	64.0 (63.9 to 64.2)	91.8 (91.7 to 92.0)	1.22 (11.39)	9.00 (7.09)	1.99 (1.92)	67.9 (67.8 to 68.1)	92.8 (92.7 to 92.9)	−1.43 (10.96)	8.48 (7.08)	1.93 (2.07)
	0–5	72.7 (72.7 to 72.8)	96.2 (96.2 to 96.3)	0.25 (9.60)	7.47 (6.04)	1.13 (1.19)	74.0 (73.9 to 74.0)	96.6 (96.6 to 96.6)	−0.96 (9.33)	7.23 (5.98)	1.10 (1.22)
Student Health Examination Sample Survey	6	57.9 (56.8 to 58.9)	87.7 (87.0 to 88.5)	0.71 (13.00)	10.28 (7.99)	2.61 (2.76)	61.4 (60.3 to 62.4)	88.6 (87.9 to 89.3)	−2.49 (12.40)	9.76 (8.05)	2.61 (2.76)
	7	53.6 (52.7 to 54.4)	85.0 (84.4 to 85.6)	−0.09 (13.81)	10.98 (8.38)	3.14 (3.18)	57.5 (56.7 to 58.4)	86.8 (86.2 to 87.4)	−2.48 (12.97)	10.34 (8.21)	3.13 (3.16)
	8	48.5 (47.6 to 49.4)	80.8 (80.2 to 81.5)	−2.42 (15.08)	12.24 (9.14)	4.07 (4.00)	51.4 (50.6 to 52.3)	83.5 (82.9 to 84.1)	−2.23 (14.22)	11.50 (8.65)	3.96 (3.80)
	9	45.5 (44.6 to 46.4)	77.2 (76.4 to 77.9)	−4.72 (15.66)	13.15 (9.72)	4.90 (4.77)	47.6 (46.6 to 48.5)	81.4 (80.7 to 82.2)	−1.36 (14.96)	12.13 (8.86)	4.54 (4.36)
	10	44.5 (43.5 to 45.5)	74.9 (74.0 to 75.8)	−7.26 (15.30)	13.61 (10.07)	5.47 (5.18)	46.7 (45.5 to 47.8)	80.4 (79.4 to 81.3)	−1.34 (15.27)	12.46 (8.93)	4.90 (4.69)
	11	46.7 (45.1 to 48.3)	76.3 (74.9 to 77.7)	−7.78 (14.62)	13.17 (10.04)	5.42 (5.18)	48.5 (46.4 to 50.6)	80.8 (79.1 to 82.4)	−0.22 (14.98)	12.11 (8.82)	4.73 (4.58)
	12	47.1 (43.9 to 50.2)	74.2 (71.4 to 76.9)	−8.36 (14.81)	13.40 (10.47)	5.68 (5.65)	50.0 (45.4 to 54.6)	81.6 (78.1 to 85.1)	−0.18 (15.11)	12.07 (9.07)	4.83 (4.81)
	6–12	49.5 (49.1 to 49.9)	80.7 (80.4 to 81.0)	−3.10 (14.97)	12.16 (9.26)	4.16 (4.30)	52.9 (52.5 to 53.3)	84.1 (83.8 to 84.4)	−1.94 (14.02)	11.24 (8.60)	3.65 (3.63)

PW10, the percentage difference of the weight estimates within 10%; PW20, the percentage difference of the weight estimates within 20%; CI, confidence interval; MPE, mean percentage error; SD, standard deviation; MAPE, mean absolute percentage error; RMSE, root mean square error.

## Data Availability

The data presented in this study were obtained from the National Health Insurance Service (NHIS) of Korea and are available upon reasonable request to the NHIS. Due to legal and ethical restrictions, the data are not publicly accessible. Researchers may apply for access through the official NHIS data request platform (https://nhiss.nhis.or.kr).

## Supplementary Materials

The following supporting information can be downloaded at: https://www.mdpi.com/article/10.3390/children12070913/s1, Table S1: PW10 values by age group based on adjusted KPET weight estimates in the SHESS Dataset (Ages ≥6 Years).
