# Supplementary material for: Validation of the Korean Pediatric Emergency Tape with Two National Anthropometric Surveys in Korean Children
Source: Children (Basel). 2025 Jul 10;12(7):913. doi: 10.3390/children12070913 (PMC12293651; doi:10.3390/children12070913)
Supplement: Supplementary file 1 [file children-12-00913-s001.zip › children-3723853-supplementary.pdf]

Supplementary Table S1. Percentage of weight estimates within 10% of actual body weight (PW10) values by age group based on adjusted Korean Pediatric Emergency Tape (KPET) weight estimates in the Student Health Examination Sample Survey Dataset (Ages ≥6 Years)

Age (y)	BT	KPET -10%	KPET -2kg	KPET -1kg	KPET	KPET +1kg	KPET +10%
6	61.4 (60.3–62.4)	52.4 (51.3–53.5)	55.1 (54.0–56.2)	59.9 (58.8–61.0)	57.9 (56.8–58.9)	48.0 (46.9–49.1)	35.5 (34.5–36.6)
7	57.8 (56.9–58.6)	49.7 (48.8–50.5)	53.2 (52.3–54.0)	56.1 (55.2–56.9)	53.6 (52.7–54.4)	47.2 (46.4–48.1)	36.2 (35.4–37.0)
8	51.4 (50.5–52.3)	43.3 (42.4–44.1)	47.4 (46.5–48.2)	49.5 (48.6–50.4)	48.5 (47.6–49.4)	44.6 (43.7–45.5)	37.7 (36.9–38.5)
9	47.6 (46.6–48.6)	38.1 (37.2–39.0)	43.4 (42.5–44.3)	45.5 (44.6–46.4)	45.5 (44.6–46.4)	43.4 (42.5–44.3)	38.2 (37.3–39.1)
10	46.4 (45.3–47.6)	33.0 (32.1–34.0)	39.5 (38.5–40.5)	43.3 (42.2–44.3)	44.5 (43.5–45.5)	44.4 (43.4–45.5)	39.6 (38.6–40.6)
11	48.2 (46.1–50.2)	29.9 (28.4–31.4)	39.2 (37.6–40.8)	44.0 (42.4–45.6)	46.7 (45.1–48.3)	47.7 (46.1–49.3)	43.5 (41.9–45.1)
12	50.8 (46.2–55.3)	30.0 (27.2–33.0)	39.1 (36.1–42.2)	44.0 (40.9–47.1)	47.1 (43.9–50.2)	47.3 (44.1–50.4)	42.8 (39.7–46.0)
Total	52.9 (52.5–53.3)	42.3 (41.9–42.7)	47.1 (46.7–47.5)	50.2 (49.8–50.6)	49.5 (49.1–49.9)	45.6 (45.2–46.0)	37.9 (37.5–38.3)

BT, Broselow Tape; KPET, Korean Pediatric Emergency Tape; PW10, Percentage Within 10% of actual weight.

Values are reported as percentages with 95% confidence intervals in parentheses.

All comparisons between the original KPET and each adjusted version were statistically significant ($p < 0.05$).
